# Virucidal Activity of Over-the-Counter Oral Care Products Against SARS-CoV-2

**DOI:** 10.3290/j.ohpd.b2960525

**Published:** 2022-04-27

**Authors:** Niranjan Ramji, Benjamin Circello, J. Leslie Winston, Aaron R. Biesbrock

**Affiliations:** a Principal Scientist, Global Oral Care R&D, The Procter & Gamble Company, Mason, OH, USA. Designed and executed the study, performed data analysis, interpreted the data, edited the manuscript, approved the manuscript for submission.; b Group Scientist, Global Bioscience, The Procter & Gamble Company, Mason, OH, USA. Designed and executed the study, performed data analysis, interpreted the data, edited the manuscript, approved the manuscript for submission.; c Vice-President, Global Health Care R&D, The Procter & Gamble Company, Mason, OH, USA. Designed the study, interpreted the data, edited the manuscript, approved the manuscript for submission.; d Senior Director and Lead Life Scientist, Global Oral Care R&D, The Procter & Gamble Company, Mason, OH, USA. Designed the study, interpreted the data, edited the manuscript, approved the manuscript for submission.

**Keywords:** COVID-19, dentifrice, mouthwash, SARS-CoV-2, virucide

## Abstract

**Purpose:**

The oral cavity is an important entry point for SARS-CoV-2 infection. This study tested whether four commercially available mouthrinses and dentifrices have in vitro virucidal activity against SARS-CoV-2 (≥4 log_10_ reduction in viral titer).

**Materials and Methods:**

One part of stock SARS-CoV-2 virus plus one part 0.3 g/l bovine serum albumin were mixed with eight parts of test product solution. After 30 s for the rinses, or 60 s for the dentifrices, the mixture was quenched in an appropriate neutralizer, serially diluted, and inoculated onto Vero E6 cells to determine viral titer. Triplicate runs were performed for each test condition with appropriate controls for test product cytotoxicity, viral interference, and neutralizer effectiveness. Test products included: 1.5% hydrogen peroxide (H_2_O_2_) rinse; 0.07% cetylpyridinium chloride (CPC) rinse; 0.454% stannous fluoride (SnF_2_) dentifrice A; and 0.454% SnF_2_ dentifrice B.

**Results:**

The 1.5% H_2_O_2_ rinse, 0.07% CPC rinse, SnF2 dentifrice A, and SnF2 dentifrice B all produced > 4 log_10_ reduction in SARS-CoV-2 titer.

**Conclusion:**

All four test products displayed potent virucidal activity in vitro. Clinical studies are warranted to determine what role, if any, these oral care products might play in preventing transmission of SARS-CoV-2 or in the management of patients currently diagnosed with COVID-19 illness.

Coronavirus disease (COVID-19) is an infectious disease resulting from the SARS-CoV-2 virus. In symptomatic patients, COVID-19 illness is associated with a wide array of complaints, including flu-like respiratory illness, gastrointestinal distress, headaches, loss of taste and smell, and skin rash. Most people with COVID-19 will experience a mild to moderate disease course and recover without requiring hospitalization, but some will develop serious illness resulting in hospitalization, long-term symptoms, or death.^40^ While anyone can develop serious COVID-19 illness, those who are older and those with underlying medical conditions, e.g. cardiovascular disease, diabetes, chronic respiratory disease, or cancer, are at higher risk.^8,14,40^ As of 1 October 2021, the World Health Organization (WHO) had received reports of 233,503,524 confirmed cases of COVID-19 illness resulting in reports of 4,777,503 deaths across the globe.^39^

SARS-CoV-2 is classified as an airborne pathogen transmitted through close contact with asymptomatic, pre-symptomatic, and symptomatic infected individuals via exposure to infected droplets and aerosols.^40^ While human-to-human transmission of SARS-CoV-2 is still under active investigation, transmission of the virus has been confirmed during breathing, coughing, sneezing, and conversing in close contact (1–3 meters).^26,34^ Positive viral RNA^5,11^ as well as viable virus samples^17^ have been detected in air samples from the hospital rooms of COVID-19 patients. During infection, SARS-CoV-2, an enveloped virus containing one positive-strand RNA genome of 29.9 kb, gains entry to human epithelial cells by using spike proteins on its surface to bind angiotensin-converting enzyme 2 (ACE2).^18^ ACE2 is expressed on the surface of many types of cells in the human body, including in the heart, gut, lungs, and nasal mucosa. It has been reported that there is a markedly high expression of ACE2 on the epithelial cells of oral mucosa and tongue.^13,30,41^ Thus, it has been proposed that the oral cavity is an important entry point for infection and further spread of SARS-CoV-2 to the gastrointestinal and respiratory tracts.^28^ Indeed, SARS-CoV-2 RNA is readily detected in the saliva of infected patients even before pharyngeal or respiratory swabbing reveals conversion.^2^ Other studies have confirmed the presence of SARS-CoV-2 RNA in gingival crevicular fluid^12^ and periodontal tissue.^7^ A recent human case-control clinical study in 568 patients reported that periodontitis is associated with increased COVID-19 complications, including death (OR=8.81, 95% CI 1.00–77.7), ICU admission (OR=3.54, 95% CI 1.39-9.05), and need for assisted ventilation (OR=4.57, 95% CI 1.19–17.4).^19^ Taken together, these data support the potential role of the oral cavity in SARS-CoV-2 transmission and infection, presenting a viable target for strategies to reduce transmission risk that may include oral care products with virucidal activity.

Cetylpyridinium chloride (CPC), a common active ingredient in many commercially available mouthrinses, has been demonstrated to have virucidal activity against influenza viruses,^23,29^ hepatitis B virus,^33^ and herpes simplex virus.^1^ CPC also exhibited antiviral activity against several coronaviruses in vitro, including HCoV-229e, MERS-CoV, and HCoV-NL63.^21,35^ CPC likely promotes viral inactivation by destroying the viral capsid as well as through lysosomotropic action, which deactivates the protective lipid coating that enveloped viruses require. These functions may ultimately block viral entry into human cells.^3,27^ In terms of the relevance of these data to SARS-CoV-2, published data with commercially available mouthrinses and sprays containing various concentrations of CPC have demonstrated inactivation of SARS-CoV-2 in vitro,^15,16^ including inactivation of several newer viral variants.^24^ In addition, a recent publication reported a reduction in the salivary viral load of SARS-CoV-2 in COVID-19 patients after rinsing with a commercial CPC-containing mouthrinse.

Another common ingredient in commercially available oral care products that is under active investigation for its virucidal activity is hydrogen peroxide (H_2_O_2_). In one key study, H_2_O_2_ vapor was found to inactivate feline calicivirus (a human norovirus surrogate), human adenovirus type 1, transmissible gastroenteritis coronavirus of pigs (a SARS-CoV surrogate), avian influenza virus, and swine influenza virus.^10^ However, a recently published study of 1.5% and 3.0% H_2_O_2_ solutions detected minimal activity against SARS-CoV-2 in vitro.^4^ A second in vitro study similarly reported minimal antiviral activity to SARS-CoV-2 with 1.5% H_2_O_2_, while in contrast, essentials oils, polyvidone-iodine, and dequalinium chloride/benzalkonium chloride rinses all delivered antiviral activity with respect to SARS-CoV-2.^20^ A pilot clinical study evaluating the efficacy of 1% H_2_O_2_ on SARS-CoV-2 found no significant reduction in the intraoral viral load in SARS-CoV-2-positive subjects after rinsing.^9^ Further investigations are therefore warranted to clarify the virucidal activity of H_2_O_2_ against SARS-CoV-2 when in a mouthrinse formulation.

The most ubiquitous commercial oral care product in homes is dentifrice. Of note, many of the more recently released formulations contain ingredients that may have virucidal activity against SARS-CoV-2, including stannous fluoride (SnF_2_) and zinc salts.^36,37^ Several commercially available dentifrices are currently under study for their potential virucidal properties,^38^ but to date, there have been no published reports of their ability to inhibit or kill SARS-CoV-2. Given that there are many variables and excipients that can impact virucidal activity of a formulation, the current study was designed to test the hypothesis that two specific, commercially available mouthrinses, one containing H_2_O_2_ and one with CPC, and two specific commercially available dentifrices containing SnF2, but differing in excipients, would have meaningful virucidal activity in vitro against SARS-CoV-2 as evidenced by at least a 4 log_10_ reduction in viral titer after a standard contact time of 30 s for the rinses or 60 s for the dentifrices.

## Materials and Methods

### Testing Facility and Compliance

All virucidal efficacy suspension testing was performed between 29 January 2021 and 3 March 2021 by Microbac Laboratories (Sterling, VA, USA). Testing conformed to the European Standard EN14476:2013+A2:2019. According to this standard, a product has virucidal activity if there is at least a 4.0 log_10_ reduction in titer beyond the cytotoxicity level.

### Oral Care Products and Reagents

The following over-the-counter oral care products were manufactured and provided by Procter & Gamble (Cincinnati, OH, USA) for use as test substances in this study:

- 1.5% H_2_O_2_ oral rinse (Oral B Mouthsore Specialty Care mouthrinse)
- 0.07% CPC oral rinse (Crest Pro-Health Clean Mint Multi-Protection mouthrinse)
- 0.454% SnF_2_ dentifrice A (Crest Pro-Health Advanced Deep Clean Mint dentifrice)
- 0.454% SnF_2_ dentifrice B (Crest Pro-Health Sensitive + Enamel Shield dentifrice)

SnF_2_ dentifrices A and B differed in excipients (inactive ingredients). Both dentifrices were tested at a 25% concentration and were prepared at 125% of the target use concentration to account for the dilution of the dentifrice in the reaction mixture. Both mouthrinse products were tested at their commercially available concentration (“neat”).

The following neutralizing reagents were used in this study:

- 1.5% H_2_O_2_ oral rinse neutralizer: minimum essential medium (MEM) + 10% newborn calf serum (NCS) + 0.5% lecithin + 0.5% sodium thiosulfate + 0.5% polysorbate-80 + 0.1% catalase
- 0.07% CPC oral rinse neutralizer: MEM + 10% NCS + 0.5% lecithin + 0.5% polysorbate-80
- 0.454% SnF_2_ dentifrice A and B neutralizer: MEM + 10% NCS + 0.5% sodium thiosulfate.

Each test had its own unique control, consisting of the neutralizing reagents listed above without the active ingredient, to determine the change in viral titer.

### Inoculum Preparation

The challenge virus for virucidal efficacy suspension testing was Severe Acute Respiratory Syndrome-Related Coronavirus 2 (SARS-CoV-2). The strain was USA-WA1/2020 (NR-52281, BEI Resources; Manassas, VA, USA). The host cell line was Vero E6 cells (ATCC CRL-1586). The stock virus was prepared by infection of Vero E6 host cells. The cultures were frozen 2-3 days after infection at -60°C to -90°C. After freezing and thawing, cell-free stocks were prepared by centrifugation. The stock virus was then aliquoted and stored at -60°C or below until used in testing.

### Pre-test Cytotoxicity Evaluation

**Fig 1:**
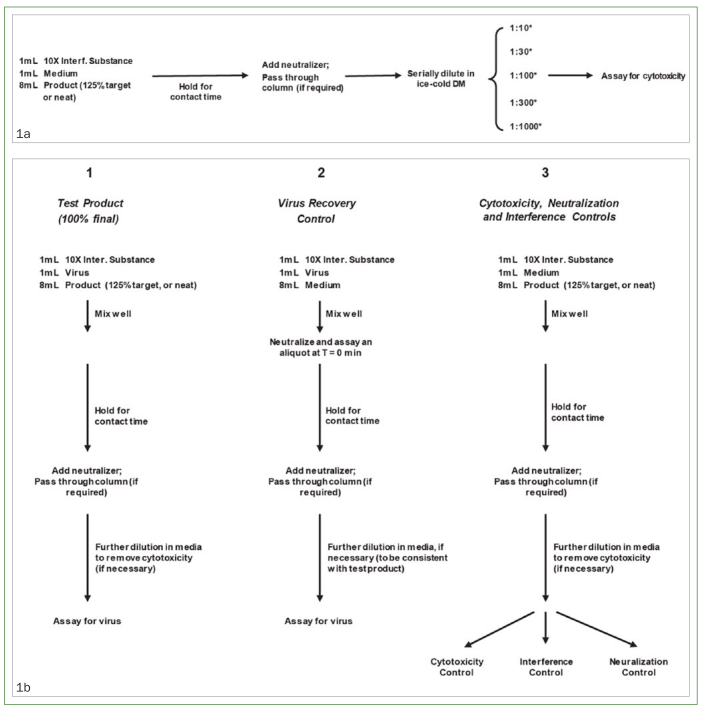
Protocols for the a) pre-test cytotoxicity evaluation and b) virucidal quantitative suspension evaluation and controls. DM: dilution medium; interfering substance: 0.3 g/l bovine serum albumin (BSA); product, mouthrinse or dentifrice test product; virus, SARS-CoV-2 stock virus.

A pre-test cytotoxicity evaluation (Fig 1A) was performed prior to virucidal efficacy suspension testing. Evaluations were run as per the virucidal efficacy suspension test; however, dilution media were used in lieu of viral suspension prior to quenching to determine appropriate dilution volumes for the test materials. The post-neutralized sample was considered undiluted; it was then diluted in ice-cold dilution medium at ratios of 1:10, 1:30, 1:100, 1:300, and 1:1000 and inoculated into eight replicate wells of the host cells for each dilution assayed. The cells were incubated at 36°C with 5 ± 3% carbon dioxide for 8 days, then evaluated for viability.

### Virucidal Efficacy Suspension Test

In triplicate, 1.0 ml of virus suspension was mixed with 1.0 ml of 0.3 g/l BSA (interfering substance). Next, 8.0 ml of the test substance was added and thoroughly mixed. The reaction mixture was held for 30 s for the rinses, or 60 s for the dentifrices, at 20 ± 2°C. Then, a 1.0-ml aliquot of the reaction mixture was drawn up and neutralized in 1.0 ml of the appropriate ice-cold neutralizing reagent. This post-neutralized sample was further quenched within 30 min with ice-cold dilution medium at a ratio of 1:100 for the mouthrinse test products and 1:10 for the dentifrice test products. Each sample was then used to make ten-fold serial dilutions in dilution medium. Host-cell plates were inoculated with 0.05 ml per well of the serial dilutions at a minimum of 8 wells per dilution, and plates were incubated at 36 ± 2°C with 5 ± 3% carbon dioxide for 8 days. After the 8-day incubation period, the plates were removed from the incubation chamber and evaluated (Fig 1B). Residual infectious virus was detected by viral-induced cytopathic effect, which is defined as cell rounding and sloughing off of the cell monolayer.

On each testing run, a viral stock titer was conducted alongside a cell viability control to demonstrate that the host cells remained viable and to confirm the sterility of the media employed throughout the incubation period.

### Virus Recovery Control

Virus recovery control was conducted to determine the recovered viral titer without exposure to the test product. These controls were performed as described for the virucidal efficacy suspension test, but neutralized immediately after product addition. This t=0 minute sample result was used as the initial viral load value in the calculation of the log_10_ reduction factor (see statistical analysis).

### Cytotoxicity, Neutralizer Effectiveness, and Viral Interference Controls

Samples were run as described for the virucidal efficacy suspension test. The resulting post-quenched samples were divided into three portions for the neutralizer effectiveness, cytotoxicity, and viral interference controls (Fig 1B).

For the cytotoxicity control, the post-quenched sample was directly inoculated onto host cells as described above for the virucidal efficacy suspension test.

The neutralizer effectiveness control ensured that residual test ingredients were not active after the appropriate neutralization procedure. For this control, 4.5 ml of the post-quenched sample was spiked with 0.5 ml of stock virus and held in an ice bath for 30 min. This was considered the 10^-1^ dilution. Selected dilutions were inoculated onto host cells as described above for the virucidal efficacy suspension test.

For the viral interference control, 0.05 ml of phosphate buffered saline and 0.05 ml of the lowest non-cytotoxic dilution of the sample were added to an appropriate number of host cell plates independently, and pre-treated for 60 min at 36 ± 2°C with 5 ± 3% CO_2_. The sample was then removed from the host-cell containing plate, and aliquots of 0.05 ml/well and 0.15 ml/well of dilution media of the 10^-3^ to 10^-8^ dilutions of the stock virus were added to the host-cell monolayer at a minimum of 8 wells per dilution. Only those dilutions of the neutralized product solution that showed a low degree of cell destruction (less than 25% of the monolayer) and produced a titer reduction of the virus of less than 1 log were used in the virucidal efficacy suspension test.

### Statistical Analysis

The 50% tissue culture infectious dose per ml (TCID_50_/ml) was determined using the Spearman-Karber method. The viral titer of each sample is reported with 95% confidence intervals following calculation via the Poisson distribution. The log_10_ reduction factor (LRF) was calculated in the following manner: log_10_ reduction factor = initial viral load (log_10_) – output viral load (log_10_). The viral load was determined in the following manner: viral load (log_10_TCID_50_) = titer (log_10_ TCID_50_/ml) + log_10_[volume (ml) x volume correction].

## Results

### Pre-test Cytotoxicity Results

The pre-test cytotoxicity control revealed that cytotoxicity was observed at dilution factors of 1:10 and 1:30 from the post-neutralized solution (see Materials and Methods) for all test products (Table 1). Only SnF_2_ dentifrice A demonstrated cytotoxicity at a dilution factor of 1:100, whereas the other test products did not. At dilutions of 1:300 and higher, none of the test products demonstrated cytotoxicity. These data allowed for appropriate dilution factors to be used during experimental testing to avoid test-product-induced cytotoxicity.

**Table 1:** Pre-test cytotoxicity control results

Dilution^a^	1.5% H_2_O_2_ mouthrinse	0.07% CPC mouthrinse	0.454% SnF_2_ dentifrice A	0.454% SnF_2_ dentifrice B
1:10	Cytotoxicity observed	Cytotoxicity observed	Cytotoxicity observed	Cytotoxicity observed
1:30	Cytotoxicity observed	Cytotoxicity observed	Cytotoxicity observed	Cytotoxicity observed
1:100	No cytotoxicity observed	No cytotoxicity observed	Cytotoxicity observed	No cytotoxicity observed
1:300	No cytotoxicity observed	No cytotoxicity observed	No cytotoxicity observed	No cytotoxicity observed
1:1000	No cytotoxicity observed	No cytotoxicity observed	No cytotoxicity observed	No cytotoxicity observed
1:3000	Not done	Not done	No cytotoxicity observed	No cytotoxicity observed

^a^ Fold-dilution from the post-neutralized solution (see Materials and Methods).

### Cytotoxicity, Neutralizer Effectiveness, and Viral Interference Control Results

In the cytotoxicity control, neither of the dentifrices at 25% concentration nor the mouthrinses at full concentration (“neat”) demonstrated any cytotoxicity to host cells in any well tested after the appropriate neutralization procedure (data not shown). The results of the neutralizer effectiveness control in which post-neutralization samples were spiked with stock virus demonstrated that the appropriate neutralization procedure for each test product did not interfere with accurate viral load assaying (Table 2). The viral interference control demonstrated that the appropriate neutralization procedure for each test product did not interfere with the infectivity of the stock SARS-CoV2 virus on host cells as compared with PBS alone (Table 3).

**Table 2:** Neutralizer effectiveness control results

Test	Viral load (log_10_ TCID_50_)
Neutralizer effectiveness control for 1.5% H_2_O_2_ mouthrinse	7.05 ± 0.22
Neutralizer effectiveness control for 0.07% CPC mouthrinse	6.80 ± 0.23
Neutralizer effectiveness control for 0.454% SnF_2_ dentifrice A	7.18 ± 0.18
Neutralizer effectiveness control for 0.454% SnF_2_ dentifrice B	7.18 ± 0.18

**Table 3:** Viral interference control results

Test Product	Concentration	Virus titer log_10_ TCID_50_/ml	Log_10_ titer difference vs control
1.5% H_2_O_2_ mouthrinse	Neat	6.55 ± 0.16	0.12
0.07% CPC mouthrinse	Neat	6.68 ± 0.20	0.25
PBS control (mouthrinse study)	N/A	6.43 ± 0.18	N/A

0.454% SnF_2_ dentifrice A	25%	6.80 ± 0.0	0.00
0.454% SnF_2_ dentifrice B	25%	6.93 ± 0.12	0.13
PBS control (dentifrice study)	N/A	6.80 ± 0.0	N/A

PBS: phosphate buffered saline.

### Virucidal Efficacy Suspension Test Results

All four test products demonstrated strong virucidal activity in the virucidal efficacy suspension test. The virucidal efficacy suspension test was run in triplicate for each test product, and the results did not differ among runs. As seen in Table 4, the 1.5% H_2_O_2_ rinse and 0.07% CPC rinse both produced a ≥4 log_10_ reduction in SARS-CoV-2 titer. Similarly, SnF_2_ dentifrice A produced a ≥4 log_10_ reduction and SnF_2_ dentifrice B produced a ≥4 log_10_ reduction in SARS-CoV-2 titer.

**Table 4:** Mouthrinse virucidal efficacy test results

Test product	Viral load^a^ (log_10_ TCID_50_)	Viral load reduction (log_10_ TCID_50_) vs virus recovery control
Virus Recovery Control (T = 0 min)	7.39 ± 0.17	N/A
1.5% H_2_O_2_ mouthrinse	≤ 3.17^b^	≥ 4.22
0.07% CPC mouthrinse	≤ 3.17^b^	≥ 4.22

^a^ Average of triplicate runs. ^b^ No virus was detected; the theoretical titer was determined based on the Poisson distribution (see Statistical Analysis).

**Table 5:** Dentifrice virucidal efficacy test results

Test product	Viral load^a^ (log_10_ TCID_50_)	Viral load reduction (log_10_ TCID_50_) vs virus recovery control
Virus recovery control (T = 0 min)	7.69 ± 0.18	N/A
0.454% SnF_2_ dentifrice A	≤ 3.65^b^	≥ 4.04
0.454% SnF_2_ dentifrice B	≤ 3.48^b^	≥ 4.21

^a^ Average of triplicate runs. ^b^ No virus was detected; the theoretical titer was determined based on the Poisson distribution (see Statistical Analysis).

## Discussion

This study adds to the growing body of evidence that certain over-the-counter, commercially available oral care products have potent virucidal activity against SARS-CoV-2, the virus responsible for COVID-19 illness. Here, our finding that the 0.07% CPC rinse produced a ≥4 log_10_ reduction in SARS-CoV-2 titer is in line with published in vitro research. Recently, Komine et al^16^ tested the in vitro virucidal activity of six commercially available mouthrinse products containing between 0.125% and 0.30% CPC and found that all of the products inactivated SARS-CoV-2 with greater than a 4 log_10_ reduction in titer. On the other hand, the 1.5% H_2_O_2_ rinse tested in this study produced a ≥4 log_10_ reduction in SARS-CoV-2 titer, which is a more robust outcome than those seen in other in vitro studies. For example, Koch-Heier et al^15^ reported that a mouthrinse containing 0.05% CPC and 1.5% H_2_O_2_ and a rinse with 0.1% chlorhexidine, 0.05% CPC, and 0.005% sodium fluoride, without ethanol, produced in vitro virucidal activity against SARS-CoV-2 (≥1.9 log_10_ and ≥2.0 log_10_ reduction in titer, respectively). A control solution of 0.05% CPC and a second control solution combining 0.05% CPC with 0.1% chlorhexidine also both demonstrated virucidal activity against SARS-CoV-2, albeit less powerfully (≥0.7 log_10_ and ≥1.2 log_10_ reduction in titer, respectively). However, neither a control 0.1% chlorhexidine solution nor a control 1.5% H_2_O_2_ solution demonstrated any significant virucidal activity in the same assay as compared with dilution medium only. The authors concluded that it was the CPC present in both of the commercial mouthrinses that produced the antiviral activity as opposed to the chlorhexidine or hydrogen peroxide.

Clinical studies of different mouthrinse formulations containing CPC or H_2_O_2_, on the other hand, have been more reflective of the findings in our study. Seneviratne et al^32^ recently conducted a clinical trial to evaluate the in vivo efficacy of three mouthrinse formulations to reduce the SARS-CoV-2 viral titer in the saliva of 36 COVID-19 patients at 5 min, 3 h, and 6 h after rinsing. The products included a 0.5% w/v povidone–iodine rinse, 0.2% w/v chlorhexidine rinse, and 0.075% CPC rinse. The CPC-containing mouthrinse produced a significant reduction in viral load as assessed by the cyclic threshold value of RT-PCR assay in the saliva of COVID-19 patients at 5 min and 6 h post-rinse compared with a water-rinse control. The rinse containing povidone-iodine also produced a significant reduction in viral load compared with a water-rinse control, but only at 6 h post-rinse. No significant reduction in viral load was seen for the chlorhexidine-containing rinse group compared to the water control. In another study, Eduardo et al^6^ tested three commercially available mouthrinses in 60 hospitalized patients with COVID-19. Products included 1) 0.075% CPC and 0.28% zinc lactate rinse; 2) 1.5% H_2_O_2_ rinse; and 3) 0.12% chlorhexidine gluconate rinse. Saliva samples were collected before rinsing, immediately after rinsing, 30 min after rinsing, and 60 min after rinsing, and the salivary SARS-CoV-2 viral load was measured by RT-PCR. They found that the mouthrinse containing chlorhexidine and the mouthrinse containing CPC and zinc lactate both significantly reduced the SARS-CoV-2 viral load at all timepoints tested. The mouthrinse with hydrogen peroxide resulted in a significant reduction in viral load but only up to 30 min after rinsing; this affect was no longer apparent at 60 min post rinse.

The variations in the in vitro and in vivo data for various mouthrinse active ingredients suggest that formulation variables and excipients may strongly impact the virucidal activity of a particular formulation, and active ingredients alone may not be fully predictive of outcome. In the current study, the 1.5% H_2_O_2_ rinse demonstrated virucidal activity equal to that of the 0.07% CPC rinse. Formulation parameters of the H_2_O_2_ rinse, such as the inclusion of polyphosphate (e.g. sodium hexametaphosphate) and acidification of peroxide, may have impacted its efficacy in the model.^22,25^ These data highlight the importance of testing specific formulations of oral care products for virucidal effects rather than generalizing based on previous work with specific active ingredients.

Both dentifrices containing 0.454% SnF_2_ that were tested in our study demonstrated robust virucidal activity (> 4.00 log_10_) against SARS-CoV-2. To our knowledge, this is the first published study to demonstrate that a dentifrice with SnF_2_ can exhibit virucidal activity against SARS-CoV-2.

Limitations of this research include exaggerated exposure time and concentration relative to in vivo usage, where dilution of rinse invariably occurs. In addition, the in vitro research experiments did not include saliva, because it has the potential to have a confounding effect on the human cell culture in the antiviral assay through intrinsic enzymatic activity and microbial contamination. Importantly, bovine submaxillary mucin was added to the assays to mimic human salivary molecules and make the assay more generalizable to the in vivo setting. Another limitation of this research is that it examined the antiviral effects in a single SARs-CoV-2 strain. However, it is important to recognize that the antiviral effect seems to be broadly conserved across the human coronavirus family. In the case of CPC, the in vitro antiviral efficacy has been confirmed against SARS-CoV-2 USA-WA1/2000, MERS-CoV, HCOV-NL63, and HCoV-229e supporting that the destruction of the viral capsid by this active is broadly conserved.^21,35^ Given that SARS-CoV-2 colonizes in the oral cavity and that oral mouthrinses and dentifrices have been shown to provide in vitro antiviral activity, it is tempting to speculate on the role of oral hygiene products as first line prevention modalities for SARs-CoV-2 prevention. However, the current data does not inform whether the use of commercially available oral care products prevent infection with SARS-CoV-2 nor lessen the severity of disease in those already infected. In vivo clinical evaluations of the virucidal efficacy of the tested products would be necessary to determine the duration of viral reduction in the oral cavity and whether product usage has any impact upon disease transmission or severity.

## Conclusion

The current study demonstrates that a 1.5% H_2_O_2_ rinse and a 0.07% CPC rinse can produce a ≥4 log_10_ reduction in SARS-CoV-2 titer in vitro after 30 s of contact time. Similarly, both 0.454% SnF_2_ dentifrice A and 0.454% SnF_2_ dentifrice B can produce a ≥4 log_10_ reduction in SARS-CoV-2 titer in vitro after 60 s of contact time. While the clinical and epidemiological implications of these data are not known, these data highlight the importance of testing specific formulations of oral care products for virucidal, antiviral, or other clinical effects rather than generalizing based on previously published in vitro or in vivo studies with specific active ingredients.
